# Predicting procedural pain after ureteroscopy: does hydrodistention play a role?

**DOI:** 10.1590/S1677-5538.IBJU.2015.0275

**Published:** 2016

**Authors:** Zeynep Gul, Kareem Alazem, Ina Li, Manoj Monga

**Affiliations:** 1 Case Western Reserve University School of Medicine, Cleveland, Ohio, United States; 2 The Cleveland Clinic, Glickman Urological and Kidney Institute, Cleveland, Ohio, United States

**Keywords:** Pain, Postoperative Period, Ureteroscopy, Ureteral Calculi, Kidney Calculi, Surgical Procedures, Operative

## Abstract

**Purpose:**

To identify perioperative predictors of immediate pain after ureteroscopy, specifically evaluating the impact of hydrodistention from irrigation on pain.

**Materials and Methods:**

We retrospectively identified patients who underwent ureteroscopy for the treatment of calculi. Data recorded for these patients included their maximum pain score in the post-anesthesia care unit (PACU), average flow rate of irrigant used during the procedure, patient and stone characteristics, operative procedure, and details of patients' immediate, post-operative course. Spearman's rho was used to determine the relationship between non-parametric, continuous variables. Then, a linear regression was performed to assess which variables could predict the peak pain score.

**Results:**

A total of 131 patients were included in the study. A non-parametric correlation analysis revealed that maximum pain score was negatively correlated with being male (r = −0.18, p=0.04), age (r = −0.34, p<0.001), and post-op foley placement (r = −0.20, p=0.02) but positively correlated with the preoperative pain score (r = 0.41, p<0.001), time in the PACU (r = 0.19, p = 0.03), and the morphine equivalent dose (MED) of narcotics administered in the PACU (r = 0.67, p<0.001). On linear regression, the significant variables were age, preoperative pain score, and stent placement. For every ten-year increase in age post-operative pain score decreased by 4/10 of a point (p = 0.03). For every 1 point increase in preoperative pain score there was a 3/10 of a point increase in the maximum pain score (p = 0.01), and leaving a stent in place post-operatively was associated with a 1.6 point increase in the maximum pain score.

**Conclusions:**

Hydrodistention does not play a role in post-ureteroscopy pain. Patients who are younger, have higher preoperative pain scores, or who are stented will experience more post-operative pain after ureteroscopy.

## INTRODUCTION

Pain is the most common complication following ureteroscopy as well as the most common reason for hospital admission after the procedure (1-3). The cause of this post-operative pain has been studied extensively but has yet to be determined. In general, pain of renal origin is thought to be secondary to distention of the renal capsule and the overlying nerve endings (4, 5). Studies from the 1950s showed that increasing pressures in or near the renal pelvis caused increased pain (6). Other potential sources of pain include ureteral spasm (7, 8), mucosal irritation, ischemia, inflammation, and activation of chemoreceptors (9, 10).

During ureteroscopy, forced irrigation causes distention of the renal collecting system. We have empirically observed petechial hemorrhage of the urothelium with forced irrigation of the upper tract; similar to what is seen with hydrodistention of the bladder. Therefore, we hypothesized that more irrigation would lead to more hydrodistention and more pain. To this end, we evaluated the impact of irrigant flow rate on post-ureteroscopy in patients who were treated for calculi. We also examined which factors, if any, were predictive of immediate post-operative pain.

## MATERIALS AND METHODS

After institutional review board approval, we retrospectively identified patients who underwent ureteroscopic removal of calculi (CPT 52351-52354, 52344). Patients and stone characteristics were obtained from the electronic medical record. Stone size was defined as the maximum diameter of the largest stone measured by preoperative CT scan, which was used also to document the stone location.

All procedures were performed by one of two endourologists at our institution, and general endotracheal intubation with inhalation agents was used for all cases. Semi-rigid ureteroscopy was utilized for distal and mid-ureteral pathology, while flexible ureteroscopy with the routine use of a ureteral access sheath was used for proximal ureteral and intrarenal pathology. Both surgeons employed a fragmentation and basketing strategy as opposed to dusting. Depending on surgeon preference, pressurized irrigation using a hand-held syringe (Boston Scientific Single-Action Pump System) or pressurized bags (100-200mmHg) was utilized for all procedures. The number of 3000mL irrigation bags used during each procedure was determined from patient-billing records.

Pain scores were quantified by patients, using a self-administered visual pain analog table. The preoperative pain score was defined as the last recorded pain score before the patient entered the operating room. The maximum pain score was the highest pain score the patient reported while in the Post-Anesthesia Care Unit (PACU). In the PACU, patients were queried by the nurses regarding their pain, starting from when they first gained consciousness, and were reassessed at regular intervals or after the administration of medications.

Patients who underwent additional procedures at the time of ureteroscopy, with the exception of cystoscopy or retrograde pyelograms, were excluded from the study. Total procedure time was defined as the time from the insertion of the cystoscope to the insertion of the postoperative urethral catheter as documented in the electronic medical record. To better approximate the pressures inside the renal collecting system during the procedure, we created a predictor variable, flow, defined as the volume of fluid used per minute.

Results were expressed as a proportion or as the mean and standard deviation. Spearman's test was used to determine the relationship between non-parametric, continuous variables. Then, a linear regression was performed to assess which variables could predict peak pain score. Statistical analysis was performed with SPSS, version 22.0 (SPSS Inc., Chicago, IL) and the significance level was set at p<0.05.

## RESULTS

A total of 131 patients were included in this study. There was an equal gender distribution (50% male). The mean age was 54±15 years and the mean BMI was 31±7.6kg/m^2^. The average diameter of the largest stone was 7.2±3.7mm and 43% of these were in the ureter. A more detailed description of patient and stone characteristics can be found in Table-1. The average flow of irrigant was 130±76mL/min and 84% of patients were stented. The average maximum pain score was 3.7±3.1 and ranged from 0-10 (Table-2). A non-parametric correlation analysis revealed that the maximum pain score was negatively correlated with being male (r=-0.18, p=0.04), age (r=-0.34, p<0.001), and post-op Foley placement (r=-0.20, p=0.02) but positively correlated with both the preoperative pain score (r=0.41, p<0.001), time in the PACU (r=0.19, p=0.03), and the morphine equivalent dose (MED) of narcotics administered in the PACU (r=0.67, p<0.001).

**Table 1:** Patient and Stone Characteristics.

		Stone Patients (n=134)			
		Mean±STD or Number (%)			Range
**Age**			53.4±14.6		21-87
	20-40		30 (22.4)		
	41-60		57 (42.5)		
	>60		47 (35.1)		
Gender (male)			50%		
BMI (kg/m^2^)		30.8		7.65	18.8-59
Preadmission narcotic use		50 (37)			
History of ureteroscopy		64 (48)			
History stent placement		79 (59)			
Stone size (mm)		7.1		3.65	1-24
**Stone location**					
	Ureter	75 (56)			
	Renal	59 (44)			
**Stone composition**					
	Calcium oxalate	89 (66.4)			
	Calcium phosphate	32 (29.3)			
	Uric Acid	6 (4.5)			
	Magnesium Ammonium Phosphate	1 (0.7)			
	Mixed	6 (4.5)			

**Table 2:** Operative Procedures, Post-Operative Exerience and Pain.

Stone Patients (n=134)				
Operative time (min)		60.3	31.7	15-192
**Procedure**				
	Ureteroscopy	28(21)		
	Nephroureteroscopy	106(79)		
No. laser used		87 (65)		
No. basket used		132(98)		
No. dilator us d		20(15)		
No. stent used		111 (83)		
**Bilateral procedures**				
	Ureteroscopy	8(6)		
	Stent Placement	4(3)		
Bags of irrigant		2.1	0.63	1-5
Preo-operative pain score		1.7	2.6	0-10
High pain score		3.8	3.1	0-10
Time in PACU (min)		199	122	47-979
Narcotics given in PACU (MEDs)		27.6	35.9	0-193

Multiple linear regression analysis was performed to identify variables that were predictive of the maximum pain score. Explanatory variables included in the model were age, sex, BMI, preoperative pain score, preadmission narcotics consumption, basket or laser use, stent placement, ureteral dilation, use of a ureteral access sheath, use of a rigid cystoscope, stone location, stone size, time spent in the PACU, narcotics administered in PACU, post-operative Foley catheter insertion, and previous history of a stone, ureteroscopy or stent. After linear regression sex, post-operative Foley placement, and time spent in the PACU were no longer significantly associated with the pain score. The variables that were found to be predictive were age, preoperative pain score, stent placement, and narcotics administered in the PACU. For every ten-year increase in age, post-operative pain score decreased by 4/10 of a point (p=0.03). For every 1 point increase in preoperative pain score there was a 3/10 of a point increase in the maximum pain score (p=0.01). Leaving a stent in place post-operatively was associated with a 1.6 point increase in the maximum post-operative pain score (p=0.05). The amount of post-operative pain correlated with the amount of post-operative narcotics required; for each MED administered in the PACU the predicted maximum pain score increased by 1/3 of a point (p<0.001) (Table-3).

**Table 3:** Linear Regression for Predictors of Post-Ureteroscopy Pain.

Patients (n=131)			
Variable	B	SE	P-value
Age	-0.39	0.17	0.03*
Sex (Male)	-0.92	0.53	0.09
BMI	0.03	0.03	0.38
Preoparative pain score	0.27	0.10	0.01*
Home narotic use	-0.28	0.54	0.60
Laser	0.10	0.66	0.88
Basket	3.43	2.00	0.08
Dilator	-0.52	0.72	0.48
Stent	1.6	0.79	0.05*
Rigid scope	-0.17	0.65	0.79
Sheath used	0.02	0.75	0.98
History of stone	-0.02	0.56	0.97
History of ureteroscopy	0.02	0.67	0.98
History of stent placement	-0.73	0.59	0.22
Diameter of largest stone	-0.02	0.07	0.74
Locatin of largest stone (kidney)	-0.40	0.54	0.46
Flow (volume irrigant/op time)	0.00	0.00	0.50
Post-procedure foley	-0.98	0.60	0.10
Time in PACU (min)	0.00	0.00	0.43
Narcotics given in PACU (MEDs)	0.03	0.01	<0.001*

R square = 0.46

## DISCUSSION

Renal colic is thought to be caused in part by increased pressure in the renal pelvis and ureters, leading to dilation of these structures, stretching of the nerve endings, and resulting in pain (4-6, 10, 11). Indeed, pain associated with hydro-distention can be severe in patients with interstitial cystitis (12). We hypothesized that patients who received more irrigant per minute of their procedure, and therefore experienced more dilation and hydrodistention of their urinary tract, would suffer more post-operative pain.

However, we found that the rate of irrigation flowing into the collecting system during ureteroscopy was not correlated with maximum pain score. The independent predictors of maximum pain score were age, preoperative pain score, stent insertion and MEDs given. The relationship between the preoperative and the postoperative pain score is difficult to discern. It is possible that high levels of preoperative pain impact an individual's sensory perception of post-operative pain. It is also possible that those who report worse preoperative pain have a lower pain tolerance or more difficulty with pain management.

The relationship noted between MEDs administered in the PACU and postoperative pain is likely a causal one; those in more pain received more narcotics. To check the robustness of our results, we performed another regression in which MEDs administered in the PACU was used as a proxy for post-operative pain, and again flow rate was not a significant variable.

There have been other studies examining pain following ureteroscopy, including the relationship between age, stent placement and pain, with disparate results (13-15). Schuster et al. reported that stones located in the kidney and longer operative times were predictive of increased pain 48 hours after surgery but sex, stone size, stent placement, and ureteral dilation were not (1). Others, however, found a statistically significant relationship between age, basket use, stone size, operative time and post-operative pain (15, 16).

The role of stent placement in post-operative pain is particularly contested. As mentioned above, Schuster et al. did not find a relationship between pain and stent placement. Others, including Borboroglu et al., found that at 48 hour patients with stents had more overall pain than patients who were not stented. Similar to our results, however, they did not find a relationship between intraoperative ureteral dilation or laser lithotripsy and either pain or narcotic consumption (17). The dubious role of stent insertion in post-ureteroscopy pain was emphasized in a recent meta-analysis, which found that there was so much heterogeneity in the results of studies examining the relationship between stent placement and pain scores, in the immediate post-postoperative period, the data could not be pooled for analysis (18).

Our study has some limitations. We did not directly measure the pressure within the ureter and renal pelvis. Instead, we used average flow rate of irrigation during the procedure as a proxy for hydrodistention. Secondly, we were unable to isolate the volume of irrigant used for the cystoscopy portion of the procedure (placement of a safety wire) from the volume of irrigant used during ureteroscopy. In addition, since this is a retrospective analysis some confounders may exist; however, a multivariate analysis with linear regression was performed to decrease the effect of potential bias. Future prospective or randomized control trials would be useful to further examine the relationship between hydrodistention of the renal collecting system and immediate pain following ureteroscopy, looking specifically at regulated pressures during irrigation.

## Conclusions

To our knowledge, no other studies have attempted to study the relationship between hydro-distention of the renal collecting system and post-ureteroscopy pain. Hydrodistention of the kidney and ureters from pressurized irrigation during ureteroscopy is not correlated with immediate postoperative pain. On linear regression, the predictors of immediate postoperative pain after ureteroscopy were the preoperative pain score, age, and stent placement. It should be anticipated that those patients with higher levels of pain prior to ureteroscopy will require greater amounts of narcotics in the PACU. Indeed, the use of adjuncts such as parenteral ketorolac or acetaminophen may be targeted to these patients.
